# Comprehensive analysis of nine m7G-related lncRNAs as prognosis factors in tumor immune microenvironment of hepatocellular carcinoma and experimental validation

**DOI:** 10.3389/fgene.2022.929035

**Published:** 2022-08-23

**Authors:** Tao Wang, Zhijia Zhou, Xuan Wang, Liping You, Wenxuan Li, Chao Zheng, Jinghao Zhang, Lingtai Wang, Xiaoni Kong, Yueqiu Gao, Xuehua Sun

**Affiliations:** ^1^ Department of Hepatology, Shuguang Hospital Affiliated to Shanghai University of Traditional Chinese Medicine, Shanghai, China; ^2^ Central Laboratory, Shuguang Hospital Affiliated to Shanghai University of Traditional Chinese Medicine, Shanghai, China

**Keywords:** hepatocellular carcinoma, N7-methylguanosine, long noncoding RNA, model, prognosis

## Abstract

**Background:** Hepatocellular carcinoma (HCC) remains the most prevalent gastrointestinal malignancy worldwide, with robust drug resistance to therapy. N7-methylguanosine (m7G) mRNA modification has been significantly related to massive human diseases. Considering the effect of m7G-modified long non-coding RNAs (lncRNAs) in HCC progression is unknown, the study aims at investigating a prognostic signature to improve clinical outcomes for patients with HCC.

**Methods:** Two independent databases (TCGA and ICGC) were used to analyze RNAseq data of HCC patients. First, co-expression analysis was applied to obtain the m7G-related lncRNAs. Moreover, consensus clustering analysis was employed to divide HCC patients into clusters. Then, using least absolute shrinkage and selection operator-Cox regression analysis, the m7G-related lncRNA prognostic signature (m7G-LPS) was first tested in the training set and then confirmed in both the testing and ICGC sets. The expression levels of the nine lncRNAs were further confirmed *via* real-time PCR in cell lines, principal component analysis, and receiver operating characteristic curve. The m7G-LPS could divide HCC patients into two different risk groups with the optimal risk score. Then, Kaplan–Meier curves, tumor mutation burden (TMB), therapeutic effects of chemotherapy agents, and expressions of immune checkpoints were performed to further enhance the availability of immunotherapeutic treatments for HCC patients.

**Results:** A total of 1465 lncRNAs associated with the m7G genes were finally selected from the TCGA database, and through the univariate Cox regression, the expression levels of 22 m7G-related lncRNAs were concerning HCC patients’ overall survival (OS). Then, the whole patients were grouped into two subgroups, and the OS in Cluster 1 was longer than that of patients in Cluster 2. Furthermore, nine prognostic m7G-related lncRNAs were identified to conduct the m7G-LPS, which were further verified. A prognostic nomogram combined age, gender, HCC grade, stage, and m7G-LPS showed strong reliability and accuracy in predicting OS in HCC patients. Finally, immune checkpoint expression, TMB, and several chemotherapy agents were remarkably associated with risk scores. More importantly, the OS of the TMB-high patients was the worst among the four groups.

**Conclusion:** The prognostic model we established was validated by abundant algorithms, which provided a new perspective on HCC tumorigenesis and thus improved individualized treatments for patients.

## Introduction

Hepatocellular carcinoma (HCC), accounting for nearly 90% of hepatic cancer cases (McGlynn et al., 2021), is the second leading cause of cancer-related deaths in men and the sixth in women (Kong et al., 2021). It was reported that China is a liver cancer–prevalent country. Epidemiological evidence has shown that China has had 410,000 new cases and 391,000 deaths of primary liver cancer, accounting for 45.3% and 47.1% of global cases, respectively, and bearing almost half of the global burden of liver cancer (Zeng et al., 2018), over half of patients are first diagnosed with advanced HCC, and more than 70% of patients recur within five years after treatment (Llovet et al., 2021). Given the complexity and heterogeneity of the pathogenesis of HCC, serum alpha-fetoprotein, the most used tumor biomarker in clinics, has poor sensitivity and specificity (Singal et al., 2020). Thus, there is an urgent need to explore and validate prognostic markers and therapeutic targets to guide physicians for further diagnosis and evaluate prognosis accurately.

With the leap forward in high-throughput sequencing technology, the great potential of long non-coding RNAs (lncRNAs) in various human diseases and critical biological mechanisms has attracted the attention of researchers (Chen K. et al., 2021). Studies showed that lncRNAs played a variety of roles in cancers, including epigenetics, DNA damage and cell cycle regulation, regulation of microRNAs, involvement in signaling pathways, and mediating hormone-induced cancers (Winkle et al., 2021). Among the RNA modifications, N7-methylguanosine (m7G) modification is ubiquitous in eubacteria, eukaryotes, and a few archaea. As the most abundant apparent modification in the 5’ cap of mRNA (Lin et al., 2018), changes in the expression of m7G modifying enzymes regulate downstream oncogenes or by altering mRNA methylation level of tumor-related genes, suggesting that studying RNA epigenetic modifications will greatly help us to unravel the unknown mysteries of tumors and open up new avenues of tumor therapy (Katsara and Schneider, 2021). Various studies showed that the aberrant m7G modifications were linked to the development and progression of numerous human cancers such as bladder cancer, lung cancer, HCC, and gastrointestinal cancers (Ying et al., 2021; Teng et al., 2021; Xia et al., 2021; Xie et al., 2020).

Accumulative work demonstrated that the dysfunctional expression of lncRNA was engaged in tumorigenesis, recurrence, and metastasis, suggesting the great potential for outcome predicting and personalized treatment guidance (Huo et al., 2017; Wei et al., 2019). Tang et al. found that three novel lncRNAs (RP11-160H22.5, XLOC-014172, and LOC149086) could act as prognostic markers for HCC metastasis (Tang et al., 2015). Shuai et al. reported that lncRNA MNX1-AS1 led to a rather poor prognosis in patients with gastric cancer (Shuai et al., 2020). Recently, Wang et al. found that m7G-related lncRNAs (LOC102554730 and LOC102555374) were significantly upregulated and aggravated disease progression in a mouse model of hypoxia-induced pulmonary hypertension (Wang et al., 2022). Nevertheless, whether and how m7G modification-related lncRNAs affect the pathogenesis of HCC remains to be elucidated. As a result, it was imperative to identify m7G-associated lncRNAs biomarkers for unraveling the mechanism and driving events of hepatocarcinogenesis from a multi-dimensional, multi-omics, and multi-systemic perspective and to develop new precision treatment strategies. Here, we reported the establishment of m7G-related lncRNA prognostic signatures (m7G-LPS) by bioinformatic and statistical analysis to predict survival outcomes in HCC patients.

## Material and methods

### Acquisition and preprocessing of hepatocellular carcinoma data

HCC samples with clinical data and normalized gene sequencing were downloaded from the publicly available databases: The Cancer Genome Atlas (TCGA; https://portal.gdc.cancer.gov) and the International Cancer Genome Consortium (ICGC) website (https://dcc.icgc.org/releases/current/Projects/LIRI-JP). In total, 374 and 230 malignant tissues of primary HCC from TCGA and ICGC with their clinical information were acquired (Supplementary Table S1). A total of 29 m7G regulators based on the published literature (Tomikawa.2018; Chen et al., 2022) and the Molecular Signatures Database (MSigDB) Team (http://www.broad.mit.edu/gsea/msigdb/) are shown in Supplementary Table S2.

### Bioinformatic analysis

Pearson correlation analysis (|coefficients| >0.4, and *p* < 0.001) and univariate Cox regression analysis were used to select lncRNAs associated with m7G genes as m7G-related lncRNAs.

Then, different HCC patients were clustered *via* the Consensus ClusterPlus package. CIBERSORT is a tool for providing gene expression feature sets for 22 immune cell subtypes. The ESTIMATE package uses the single sample gene set enrichment analysis (ssGSEA) algorithm to score individual samples of both stromal and immune gene sets in the tumor expression matrix and, thus, the content of these two types of cells, and finally, calculate the tumor purity.

The m7G-LPS was established *via* the least absolute shrinkage and selection operator (LASSO)-Cox regression. The LASSO is extensively utilized in clinical studies (Chen T. et al., 2021; Liu et al., 2021; Liu et al., 2022a; Liu et al., 2022b; Liu et al., 2022c). The risk score was calculated as Risk score = ∑ni = 1Coefi×Xi (Coef i represents the coefficient). Thus, high- and low-risk groups were divided according to the median risk score. The Kaplan–Meier (K-M) survival curve, receiver characteristic (ROC) curve, and principal component analysis (PCA) analyses were all used to comprehensively validate and compare the accuracy of the m7G-LPS model. Finally, a nomogram was established to demonstrate clinicopathological factors and risk values at 1-, 3-, and 5-years.

The Gene Set Enrichment Analysis (GSEA) displayed the biological function of the high- and low-risk groups. We analyzed the degree of immune cell infiltration and enrichment in each sample by ssGSEA. In addition, we used the pRRophetic and ggplot2 packages to assess the sensitivity to chemotherapy.

### Quantitative real-time polymerase chain reaction analysis

Normal human liver cell (L02) and HCC cell lines (Hu-7) were purchased from the Cell Bank of the Chinese Academy of Sciences (Shanghai, China). All cells were cultured in high fructose DMEM (gibco) at 37°C in a 5% CO_2_-containing humidified incubator. Total RNA of cultured cells was extracted using TRIzol reagent (Invitrogen), and cDNA was synthesized using a reverse transcription master kit (Invitrogen). Quantitative real-time polymerase chain reaction (qRT-PCR) analysis was quantified using SYBR-Green (Takara). The primer sequence is listed in Supplementary Table S3. Expression values were calculated using the 2^−ΔΔCt^ method. β-Actin was used as endogenous control.

### Statistical analysis

All biological statistical analyses in the study were performed using the R software version 4.1.2; unless otherwise specified, *p* < 0.05 was considered statistically significant.

## Results

### Expression profiles of m7G-Related in patients with hepatocellular carcinoma

**FIGURE 1:**
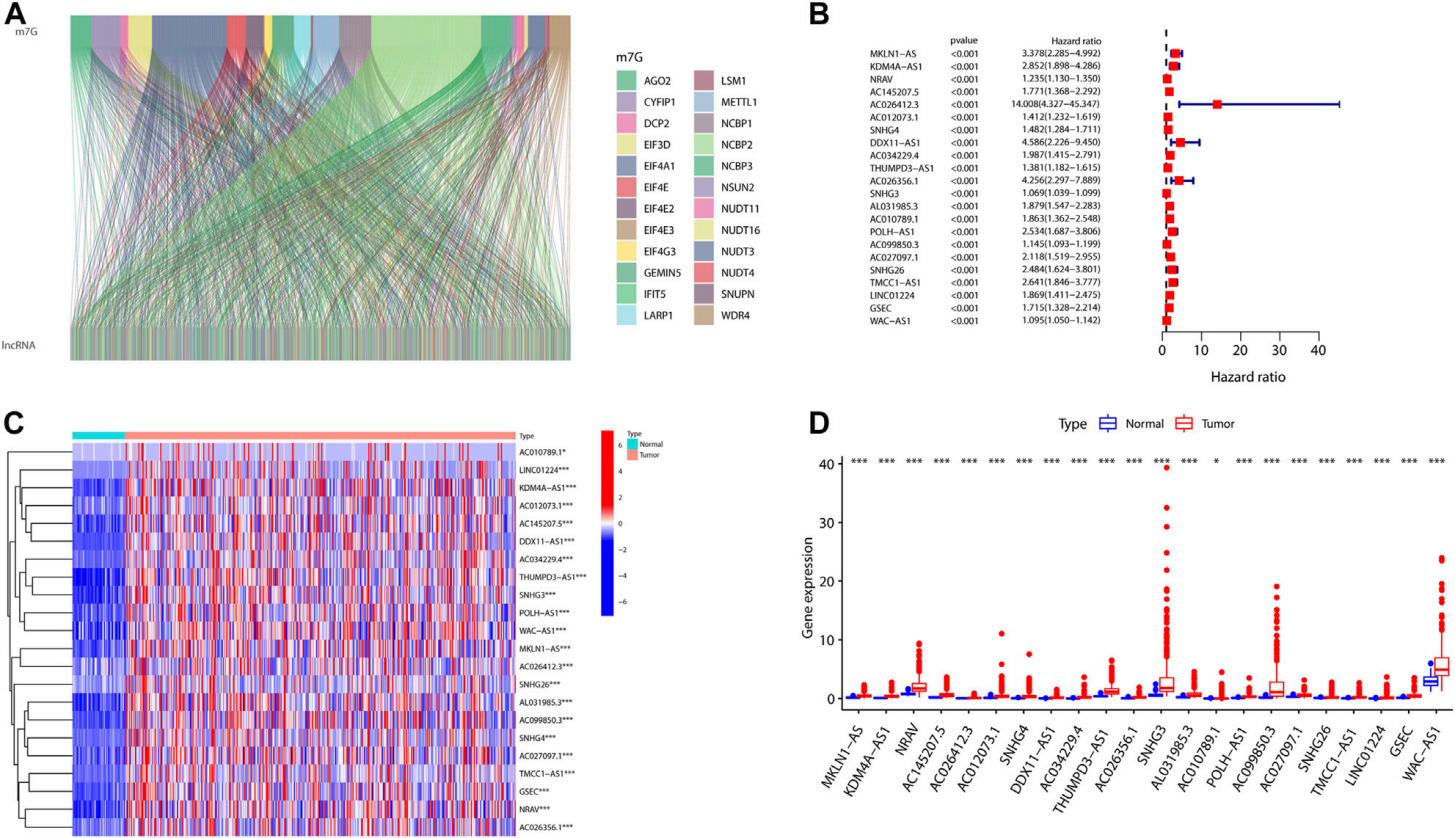
Expression profiles of m7G-related genes in patients with HCC. **(A)** Sankey for m7G-related genes and m7G-related lncRNAs. **(B)** Forest plot of 22 prognostic m7G-related lncRNAs. **(C,D)** Heatmap **(C)** and expression **(D)** of 22 m7G-related lncRNAs in the TCGA set. (*p < 0.05; ***p < 0.001). m7G, N7-methylguanosine; HCC, hepatocellular carcinoma; lncRNAs, long non-coding RNAs.

In the view of our flowchart (Supplementary Figure S1), 1465 lncRNAs were shown to be significantly corrected with 29 m7G (Figure 1A). A univariate Cox regression analysis was implemented that 22 of 1465 were defined as prognostic m7G-related lncRNAs in HCC patients (Figure 1B). We then analyzed the levels of the 22 lncRNAs in HCC patients and found that all of them were overexpressed lncRNAs (Figures 1C–D).

### Consensus clustering of prognostic m7G-Related lncRNAs

**FIGURE 2:**
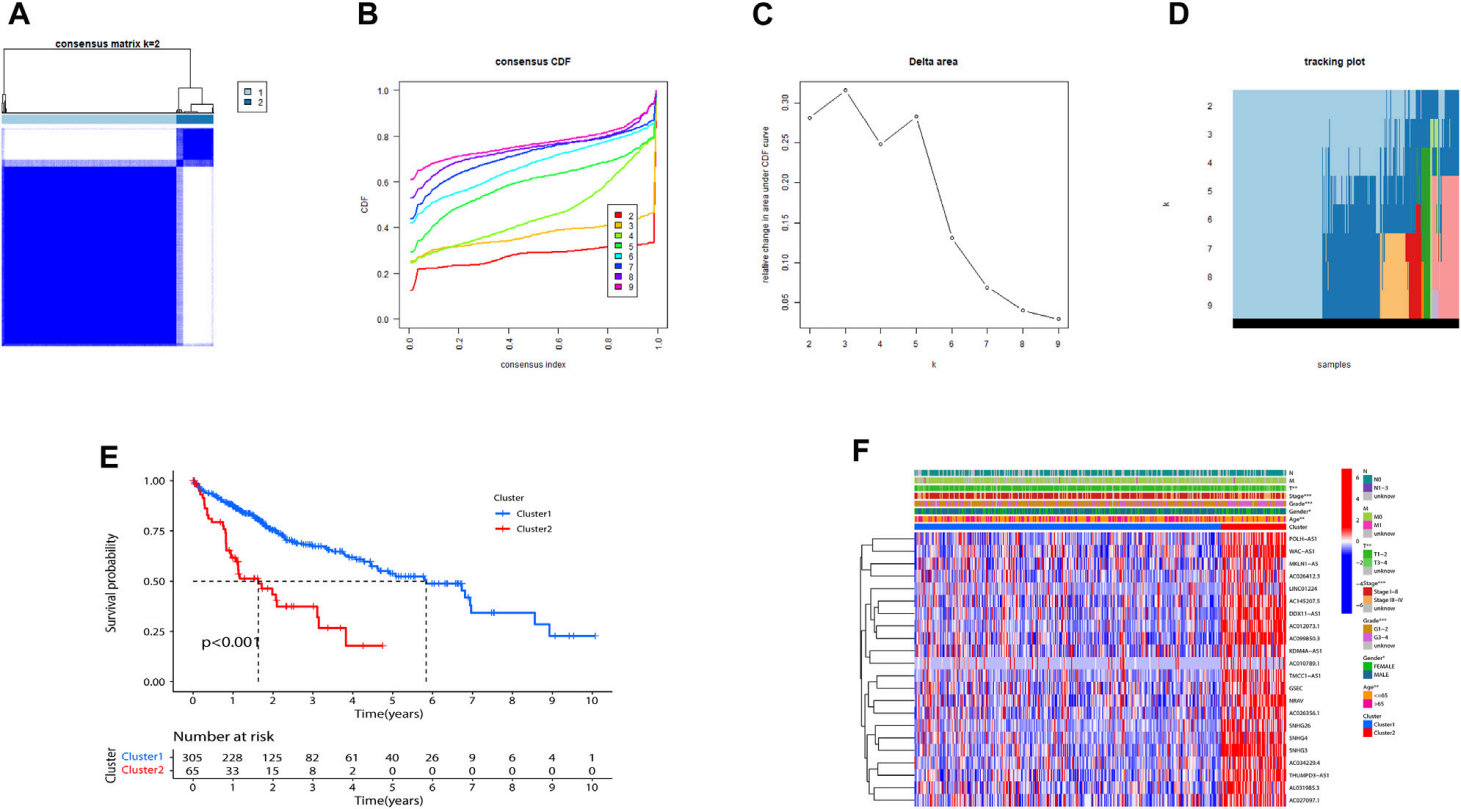
Consensus matrix of prognostic m7G-related lncRNAs. **(A–D)** Consensus clustering matrix for k = 2. **(E)** Kaplan–Meier of overall survival for the two different clusters of HCC patients. **(F)** Heatmap of Cluster1 and Cluster2 with clinicopathological features and expressions of 22 m7G-related lncRNAs in each cluster. (**p < 0.01; ***p < 0.001). m7G, N7-methylguanosine; lncRNAs, long non-coding RNAs.

Consensus clustering was utilized to classify HCC subgroups according to the expression of prognostic m7G-related lncRNAs. Finally, we selected k = 2 to minimize interference between subgroups (Figures 2A–D). Thus, two HCC subtypes: Cluster 1 (*n* = 305) and Cluster 2 (*n* = 65) were identified. Notably, the K-M curve analysis showed that Cluster1 had a longer overall survival (OS) than Cluster 2 (Figure 2E). As illustrated in Figure 2F, expressions of the 22 lncRNAs in Cluster 2 were substantially higher than that in Cluster 1. Not surprisingly, patients in Cluster 2 were more markedly related to an advanced clinical stage and grade than those in Cluster 1.

### Consensus clustering correlated with immune infiltration

**FIGURE 3:**
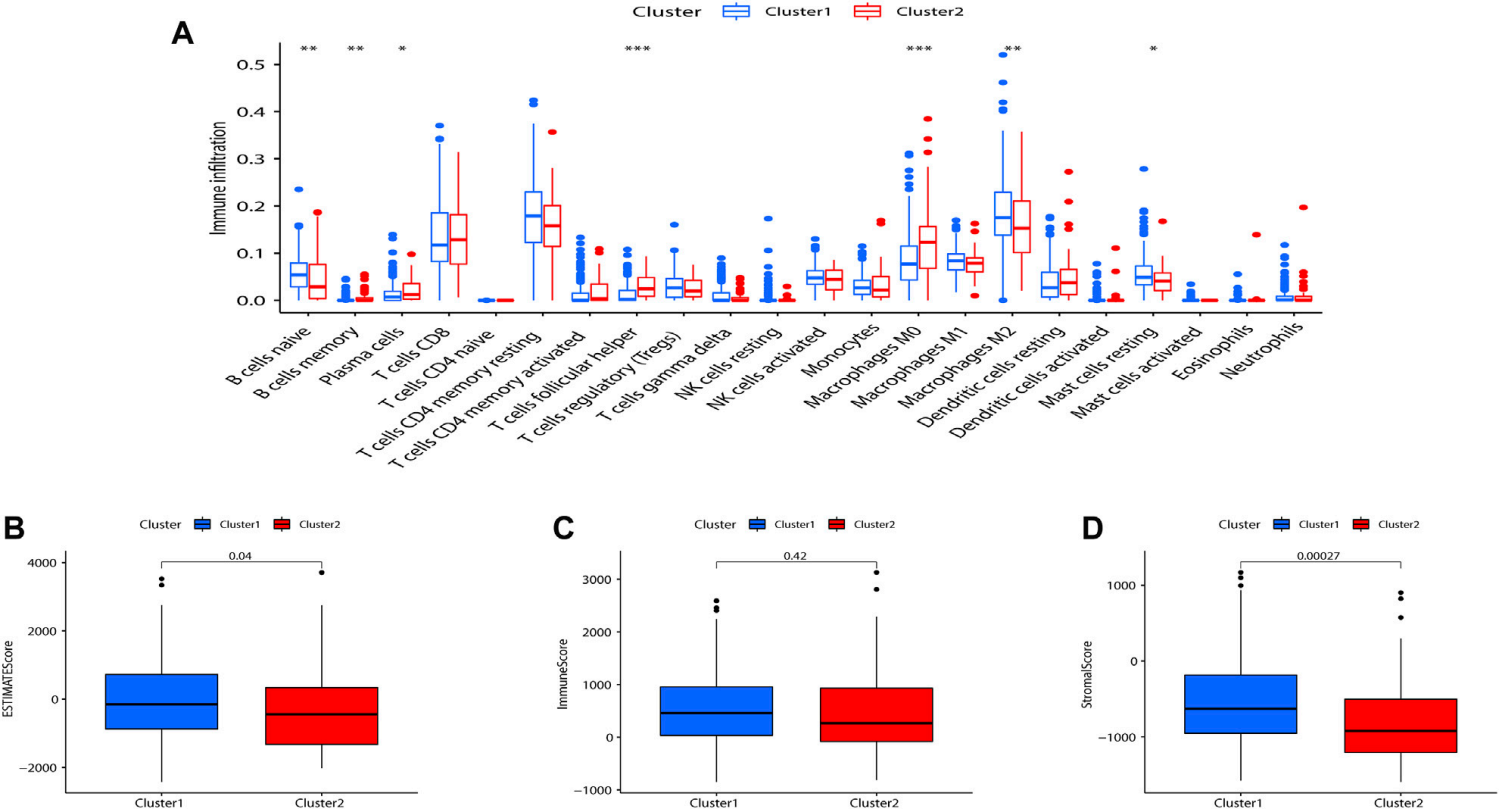
Consensus clustering correlated with immune infiltration. **(A)** Differences in the level of infiltration of 22 immune cells in the two clusters. The expression of the comprehensive score **(B)**, immune score **(C)**, and stromal cell score **(D)** between the two clusters. (*p < 0.05; **p < 0.01).

Tumor-infiltrating immune cells are instructive in evaluating tumor prognosis and immunotherapy efficacy. CIBERSORT analysis showed that compared with Cluster 2, B cells memory (*p* < 0.01), plasma cells (*p* < 0.05), T cells follicular helper (*p* < 0.001), M2 macrophages (*p* < 0.001), and mast cells resting (*p* < 0.05) were significantly upregulated in Cluster 1 (Figure 3A). The immune score, stromal score, and ESTIMATE score between the two indicated that Cluster 1 was more enriched in immune-related cells and with lower tumor cell purity compared to Cluster 2 (Figures 3B–D).

### Establishment of m7G-related lncRNA prognostic signature

**FIGURE 4:**
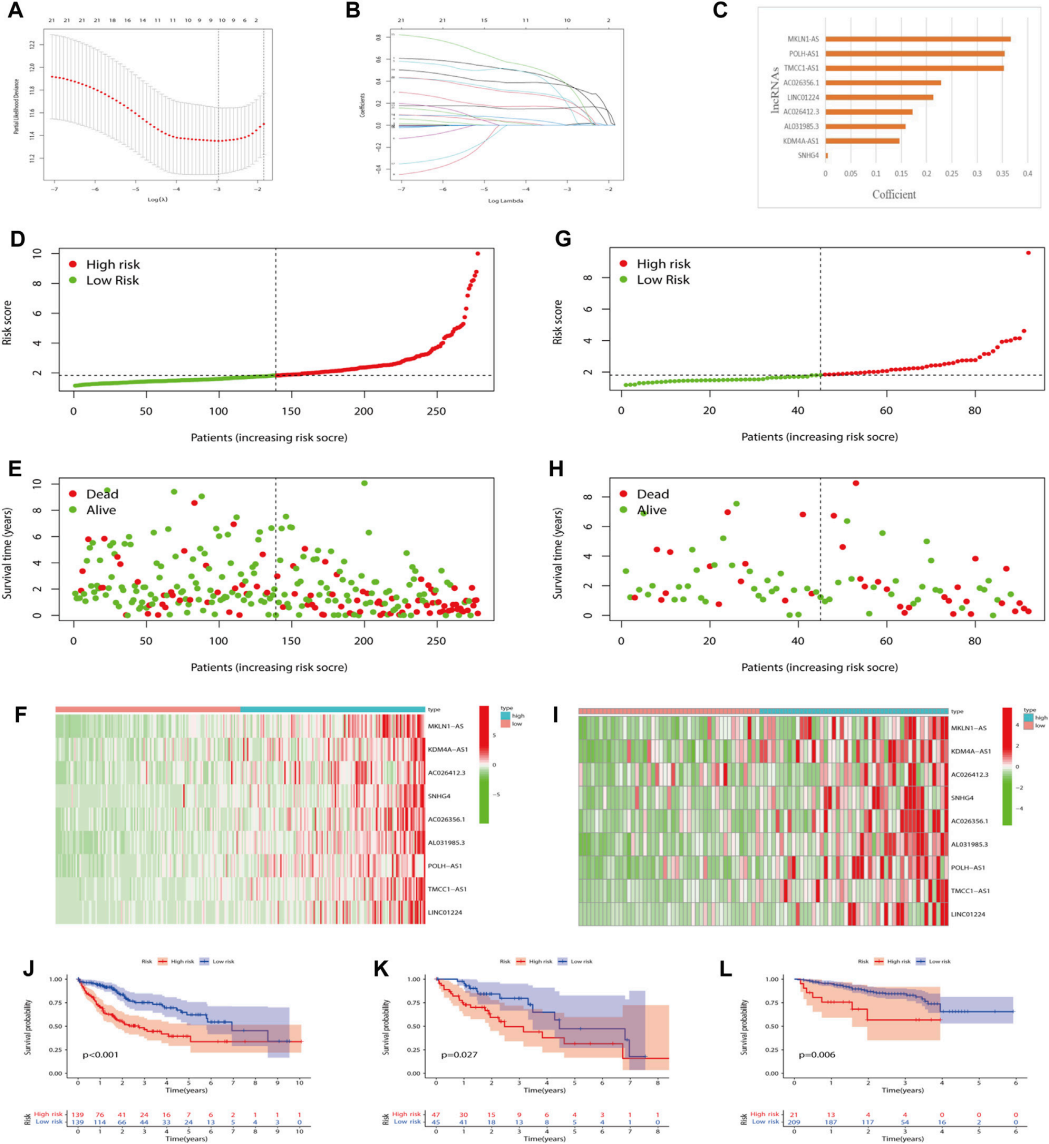
Prognostic value of the m7G-LPS. **(A–C)** LASSO regression was performed **(A)**, and the optimal criteria **(B)** and coefficients **(C)** were calculated. **(D–I)** Risk plots, survival state, and heatmap of HCC patients at low- and high-risk scores in training **(D–F)** and testing set **(G–I)**. **(J–L)** Kaplan–Meier analysis in training, testing and ICGC set. m7G, N7-methylguanosine; lncRNAs, long non-coding RNAs. m7G-LPS, m7G-related lncRNA prognostic signature; LASSO, Least absolute shrinkage and selection operator; HCC, hepatocellular carcinoma.

Nine lncRNAs were finally selected to frame a prognostic signature m7G-LPS for predicting HCC patients’ OS through LASSO-Cox regression analysis (Figures 4A–C). The m7G-LPS were constructed based on FPKM value and corresponding coefficients: risk score = (0.366825372367671 × expression of MKLN1-AS) + (0.146426813107297 × expression of KDM4A-AS1) + (0.172062395362062 × expression of AC026412.3) + (0.00427733397199361 × expression of SNHG4) + (0.228579214185232 × expression of AC026356.1) + (0.158465677466972 × expression of AL031985.3) + (0.354660948654385 × expression of POLH-AS1) + (0.353144896760336 × expression of TMCC1-AS1) + (0.213408781976956 × expression of LINC01224).

### Prognostic value of the m7G-LPS

To explore the robustness and accuracy of the m7G-LPS, we categorized 370 malignant tissues into a training set (*n* = 278) and a testing set (n = 92) according to the median risk score. The nine lncRNAs were overexpressed in high-risk groups (Figures 4D–I), and the same results were observed in the testing set (Figures 4G–I). In addition, real-time PCR results on the expression of the nine m7G-related lncRNAs were in line with our prediction results (Supplementary Figure S2). Furthermore, K-M curves showed that HCC patients with high-risk scores had worse OS than those with low-risk scores in both the training and testing sets (Figures 4J and K). Considering the heterogeneity of HCC, we choose ICGC data as an independent validation to further verify the accuracy of m7G-LPS. As expected, the result in the ICGC set including 230 HCC patients was in line with the TCGA (Figure 4L). Hence the risk score had a strong capacity to forecast the OS of HCC patients.

### Further verifies the ability of the m7G-LPS

**FIGURE 5:**
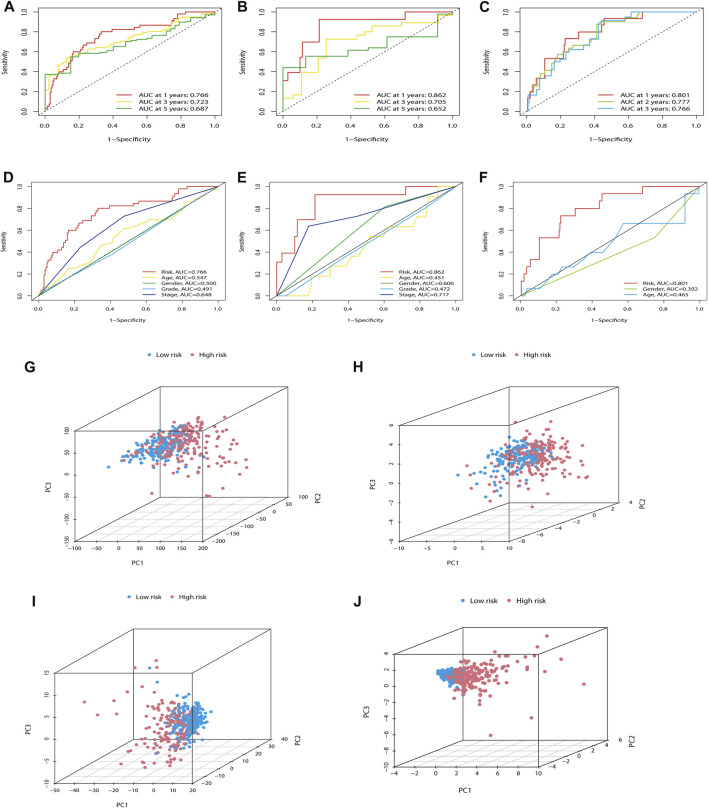
Further verification of the ability of the m7G-LPS. **(A–C)** ROC curves of m7G-PLS to predict the sensitivity and specificity of 1-, 3-, and 5-years survival in training **(A)**, testing **(B)**, and ICGC sets **(C)**. **(D–F)** The time-dependent AUC value with different clinical characteristics in the training, testing, and ICGC set. **(G–J)** PCA between the high- and low-risk groups based on whole gene expression profiles **(G)**, 29 m7G genes **(H)**, 22 prognostic m7G-related lncRNAs **(I)**, and m7G-LPS **(J)**. m7G, N7-methylguanosine; lncRNAs, long non-coding RNAs; m7G-LPS, m7G-related lncRNA prognostic signature; ROC, Receiver operating characteristic curve.

In addition, we also predicted the capability of the established m7G-LPS in the training, testing, and ICGC sets. The model showed excellent predictive accuracy (Figures 5A–C). In addition, the predictive power of the m7G-LPS was far higher than that of the clinical data across the training and testing sets, as evidenced by time-dependent ROC analysis (Figures 5D–F). PCA analysis revealed different whole gene expression profiles (Figure 5G), 29 m7G genes (Figure 5H), 22 prognostic m7G-related lncRNAs (Figure 5I), and m7G-LPS (Figure 5J). However, the results obtained based on our model illustrated a high separation between high- and low-risk groups (Figure 5J). More importantly, compared with the HCC prognostic models constructed by Zhou et al. (2021), Zhang et al. (2022a), and Jin et al. (2021), our model has a stronger predictive power (Supplementary Figures S2A–S2D). All these details certificated an excellent precision of the m7G-LPS.

### Validation of the m7G-LPS in clinical features of hepatocellular carcinoma

**FIGURE 6:**
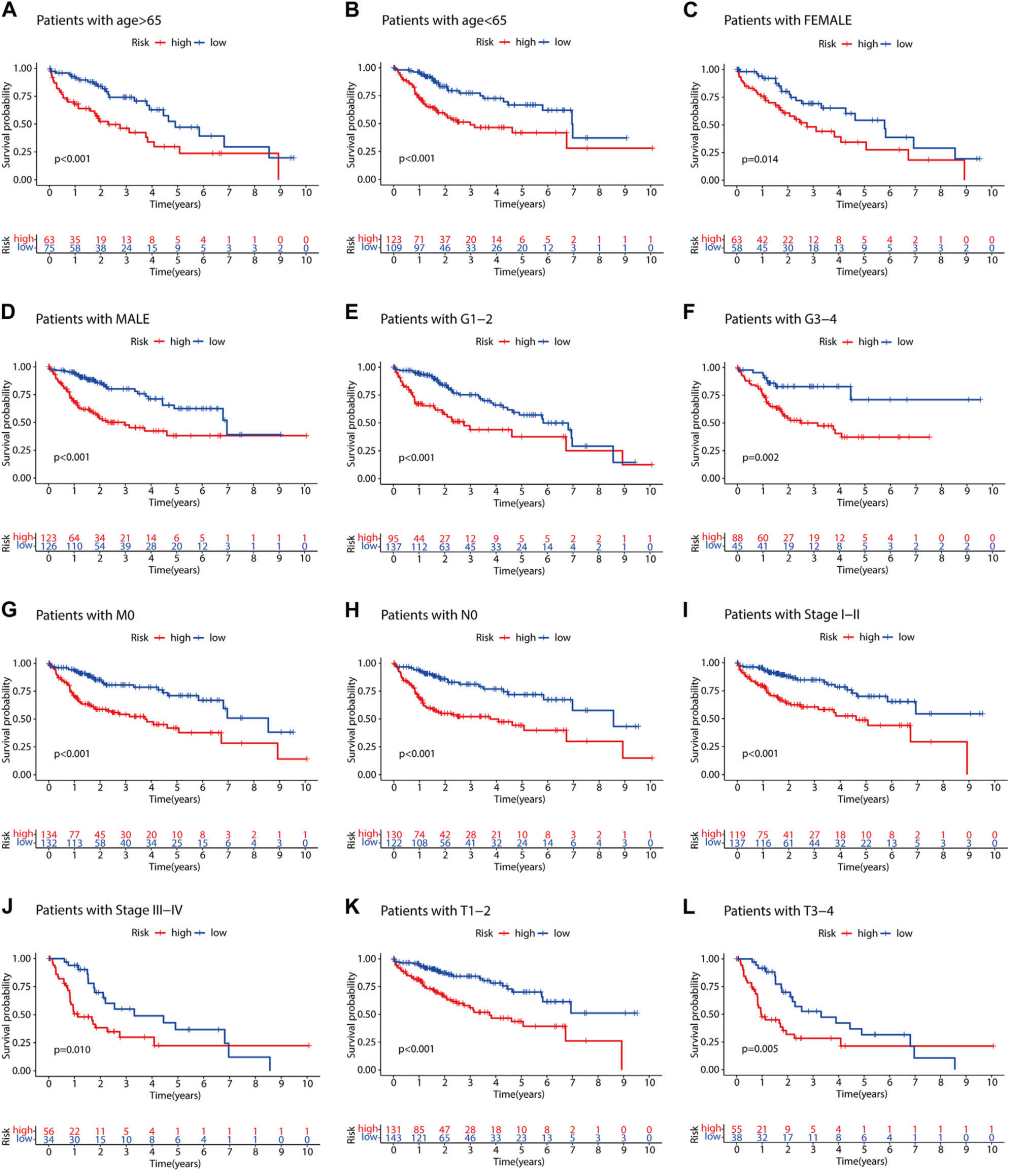
**(A–L)** Kaplan–Meier analyses of patients with AJCC-T, tumor grade, stage, gender, and age.

**FIGURE 7:**
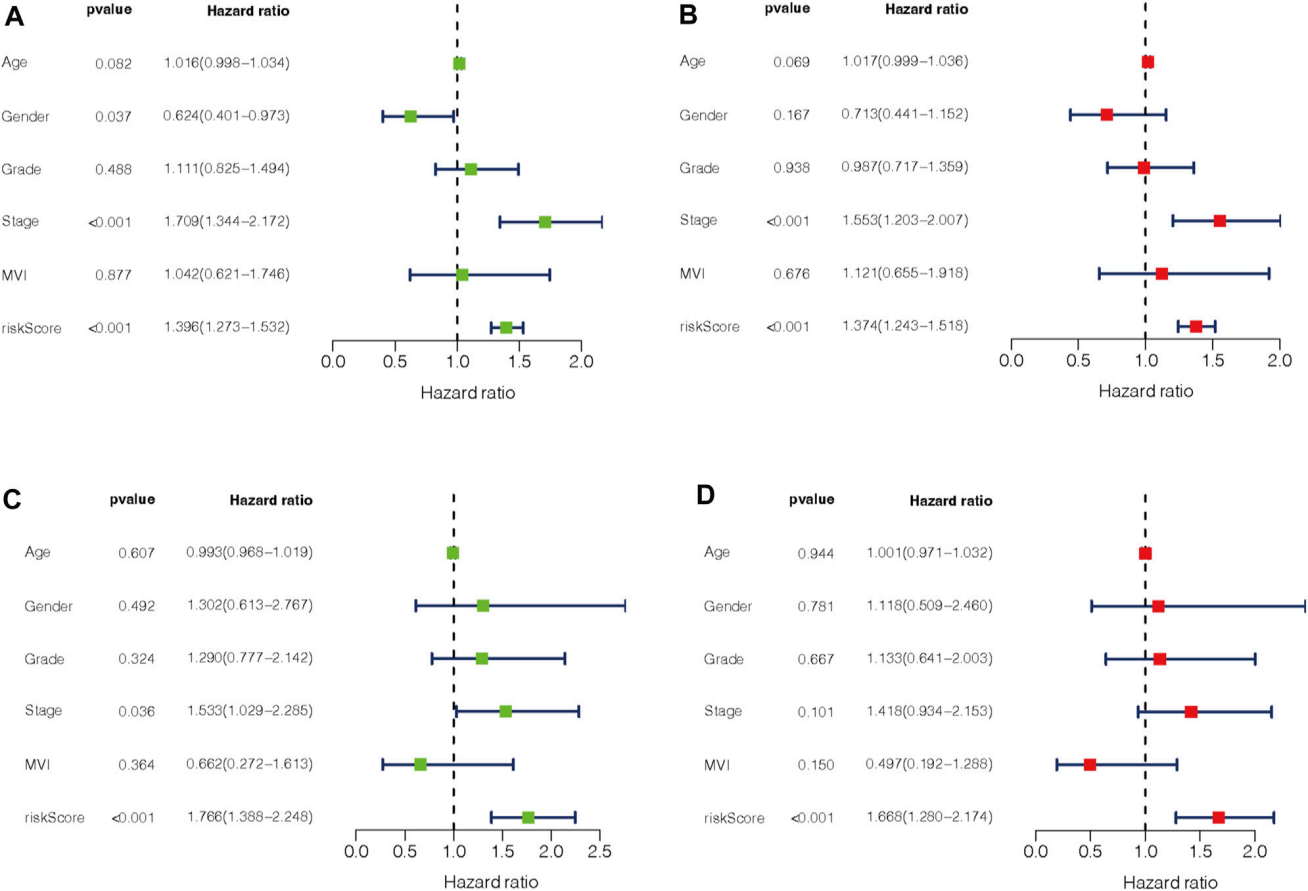
**(A–D)** Univariate and multivariate Cox regression analysis in training **(A,B)** and testing set **(C,D)**.

**FIGURE 8:**
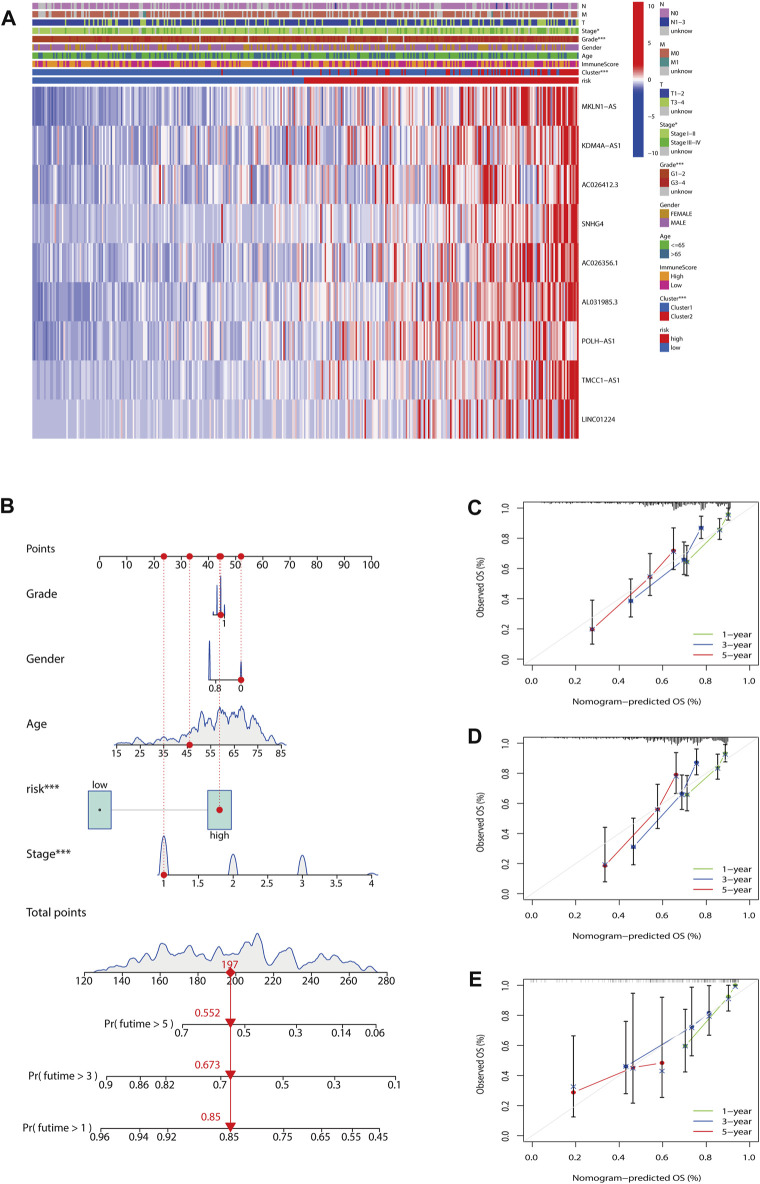
Establishment of nomogram. **(A)** The heatmap included the expression of nine m7G-LPS, clustering characteristics, immune score, and TMN stage. **(B)** Nomogram based on grade, gender, age, and risk score. **(C–E)** Calibration curves of the nomogram for predicting and observing 1-, 3-, and 5-years OS (*p < 0.05; ***p < 0.001). m7G-LPS, m7G-related lncRNA prognostic signature. m7G, N7-methylguanosine; lncRNAs, long non-coding RNAs.

To evaluate the impact of our prognostic risk model on the clinical characteristics of HCC patients, we conducted a hierarchical analysis based on their universal clinicopathological characteristics. The finding confirmed that according to the risk score, the prognosis of HCC patients could be distinguished well (Figures 6A–L). In addition, the ability of m7G-LPS was also verified by clinical features, as shown in Supplementary Figure S3. After further bioinformatics statistics, the risk score was determined as an independent prognostic symbol for HCC, irrespective of other clinical features (Figures 7A–D). The heatmap showed that grade, cluster, and stage were significant factors between the two different risk groups (Figure 8A). Cluster 2 had a high proportion in the high-risk group, indicating an unfavorable prognosis in the high-risk group. To assess the possible impact of different interventional treatment options on patients, we established a nomogram incorporating the risk score and clinical features (Figure 8B). Additionally, the results of the calibration curve analysis showed that the predicted survival probability by the nomogram was consistent with the actual one (Figures 8C–E).

### Gene set enrichment analysis

**FIGURE 9:**
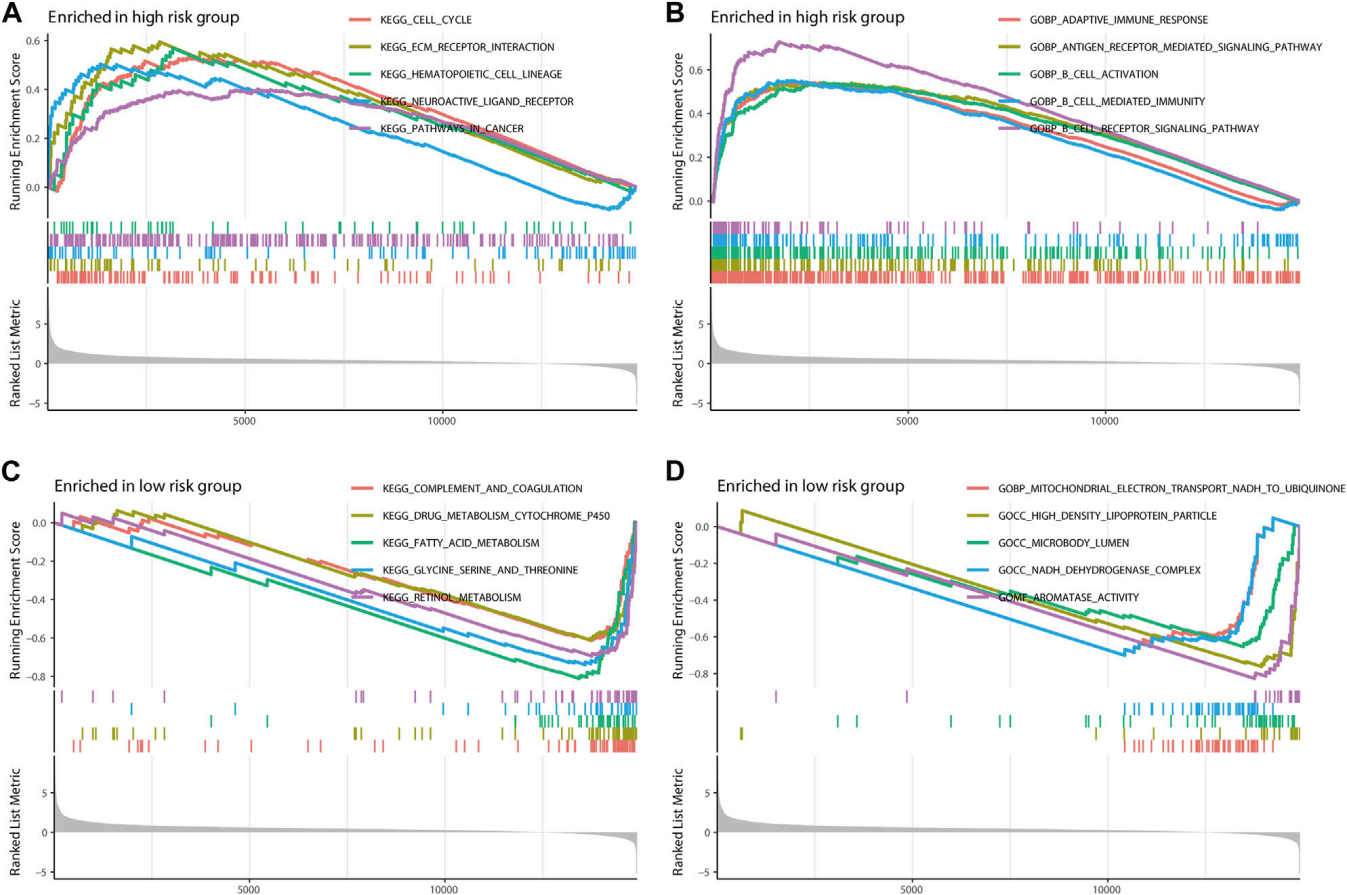
Gene Set Enrichment Analysis. The top five pathways in two different risk groups by KEGG enrichment analysis **(A,C)** and GO enrichment analysis **(B,D)**. KEGG, Kyoto Encyclopedia of Genes and Genomes; GO, Gene Ontology.

The Kyoto Encyclopedia of Genes and Genomes (KEGG) and the Gene Ontology (GO) enrichment analysis demonstrated that m7G-LPS was enriched in immune-, metabolism-, and tumor-related pathways. In the KEGG enrichment analysis, the cell cycle, extracellular matrix (ECM)-interaction, hematopoietic-cell-lineage, neuroactive-ligand receptor interaction, and pathways in cancer were significantly enriched in the high-risk group (Figure 9A). The significantly enriched pathways in the low-risk group were various metabolic processes, including drug metabolism-cytochrome-P450, fatty acid metabolism, glycine metabolism, serine metabolism, threonine metabolism, and retinal metabolism (Figure 9B). In GO enrichment analysis, the significantly enriched pathways in the high-risk group were various immune processes such as adaptive-immune response, B-cell activation, B cell-mediated immunity, and B-cell receptor signaling pathway (Figure 9C), while the GO function enrichment of low-risk score demonstrated enrichment in mitochondrial-electron-transport-NADH-to-ubiquinone, high-density-lipoprotein-particle, microbody-lumen, NADH-dehydrogenase-complex, and protein–lipid-complex (Figure 9D).

### Tumor immune cell infiltration and gene expression

**FIGURE 10:**
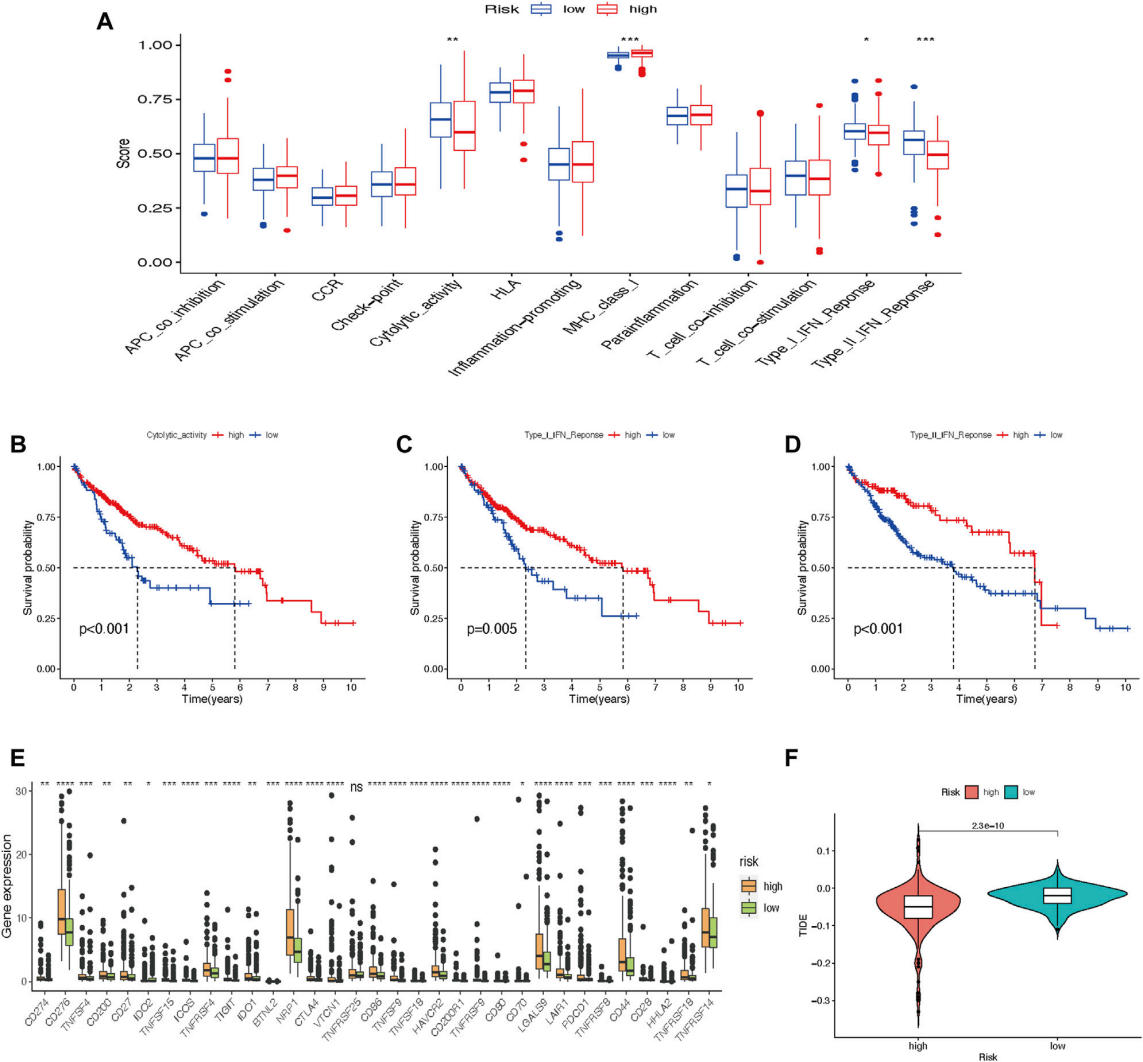
Tumor immune cell infiltration and gene expression. **(A)** Box plot of immunologic function analysis. **(B–D)** Relationship between cytolytic-activity, type-I-IFN-response, and type-II-IFN-response and survival of HCC patients in high- and low-risk groups. **(E)** Box plot of 33 immune checkpoints. **(F)** TIDE analysis shows different immune responses in high- and low-risk groups (*p < 0.05; **p < 0.01; ***p < 0.001). HCC, hepatocellular carcinoma; TIDE, tumor immune dysfunction and exclusion.

Immune cell-related functions, including cytolytic activity, major histocompatibility complex-class-I, type-I-interferon (IFN)-response, and type-II-IFN-response were remarkably different between the two risk groups (Figure 10A). We further found that cytolytic activity, type-I-IFN-response, and type-II-IFN-response were closely related to the survival of HCC patients (Figures 10B–D). In comparison with the low-risk group, immune checkpoint expressions in the high-risk group were higher (Figure 10E). Furthermore, the score of Tumor Immune Dysfunction and Exclusion (TIDE) was significantly lower in the high-risk group than in the low-risk group (Figure 10F), which suggested that patients in the high-risk subtype may have a better prospect for immunotherapy.

### Risk-related drug-sensitivity prediction

**FIGURE 11:**
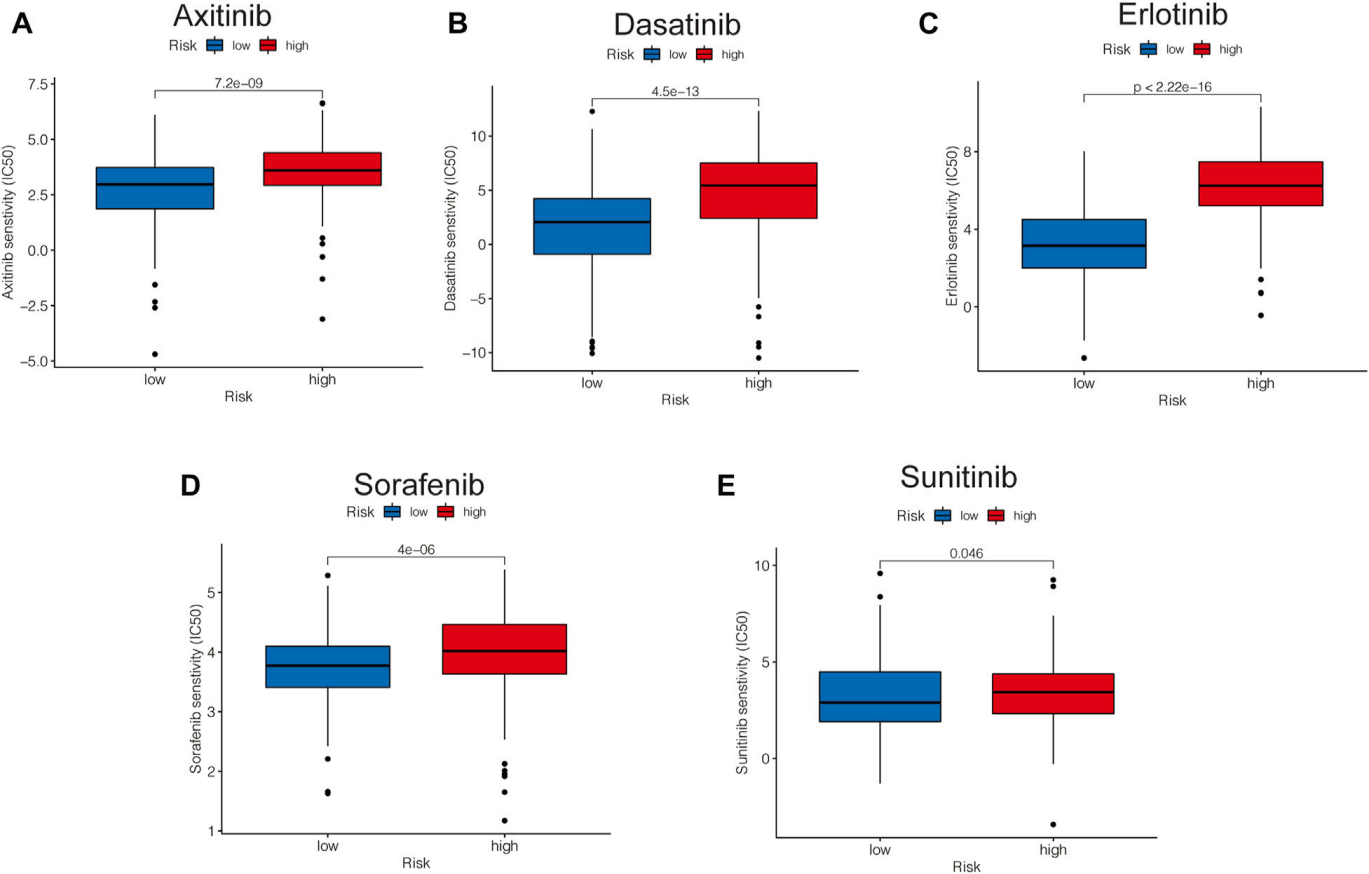
Risk-related drug-sensitivity prediction. **(A–E)** Relationship between risk score and chemotherapeutic sensitivity.

Based on the pRRophetic package, some representative and commonly used clinical drugs were predicted to have different effectiveness in HCC patients with different risk scores. The results indicated that axitinib, dasatinib, erlotinib, and sorafenib were more effective in the low-risk group, while sunitinib may be more effective in the high-risk group (Figures 11A–E).

### Correlation between the m7G-LPS and tumor mutation burden

**FIGURE 12:**
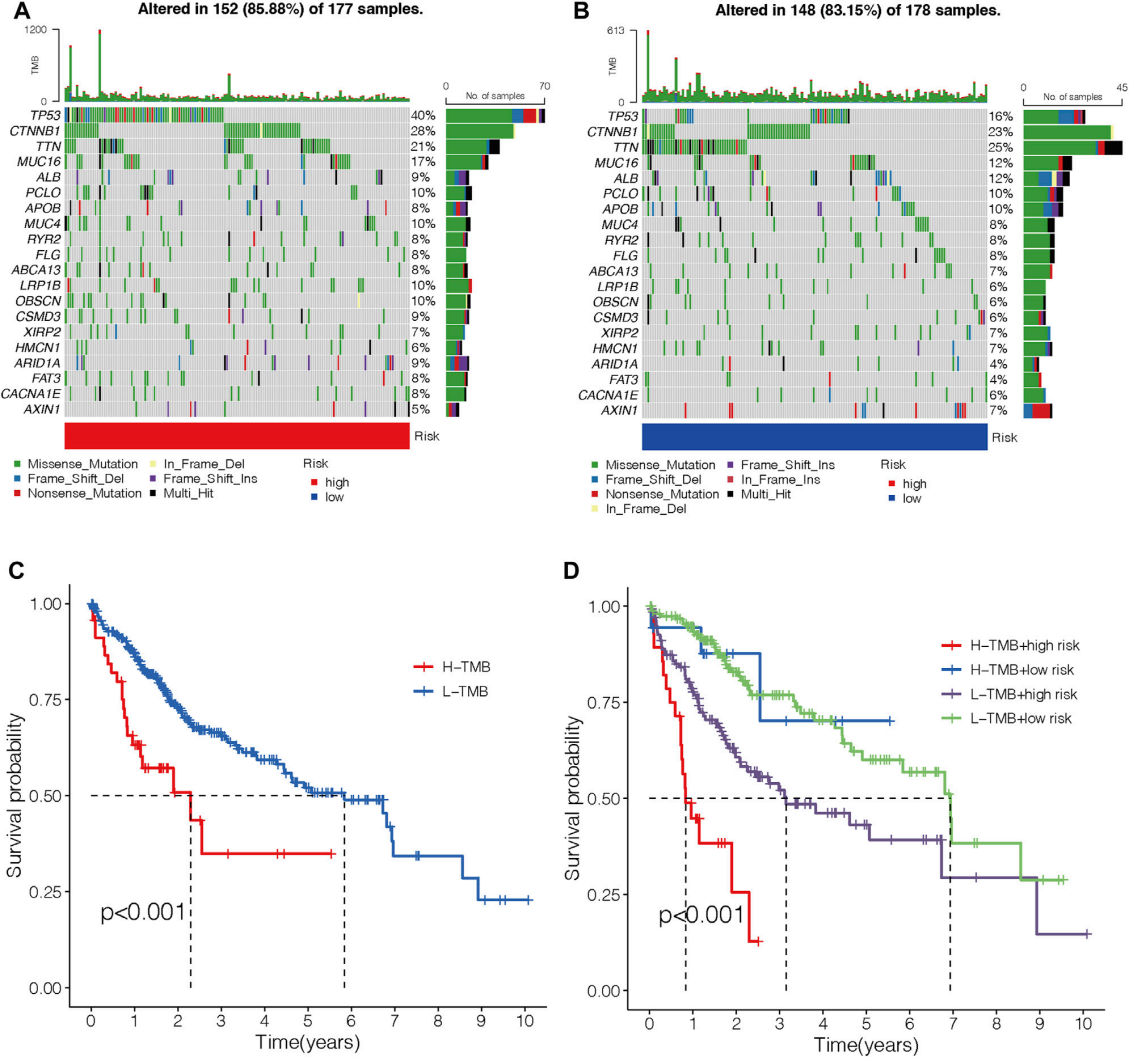
Correlations between the risk model and TMB. **(A–B)** Waterfall diagram shows the top 20 mutated genes in the high- **(A)** and low-risk groups **(B)**. **(C)** OS curves for high- and low-TMB sets. **(D)** OS curves for patients are divided into both TMB and risk scores. TMB, tumor mutation burden. OS, overall survival.

An oncoplot showed the topmost 20 mutated HCC genes in the two different risk groups (Figures 12A and B). The outcomes revealed that TP53, CTNNB1, and MUC16 were more frequently mutated in the high-risk group, while TTN, ALB, and APOB were more frequently mutated genes in the low-risk group. TMB-high HCC patients had a shorter OS (*p* < 0.001; Figure 12C). Moreover, patients with both the high TMB and the high-risk score had the worst OS among the four groups (*p* < 0.001; Figure 12D).

## Discussion

Despite some success in diagnosis and development of vaccines, the surge in HCC prevalence and short overall survival has spurred investigators to discover new prognostic signatures for HCC patients, which will enable stratification of patients and precise treatment. Since 2018, there has been a mushrooming of research studies on the processes of m6A in the proliferation, differentiation, prognosis, and apoptosis of various tumors (Yi et al., 2020; Zuo et al., 2020). Precise prognostic models help physicians make better clinical decisions and allow health authorities to allocate health care resources more rationally (Ferraro et al., 2022). However, no m7G-related lncRNAs signature has been reported in tumor research. Therefore, it is crucial to explore the m7G-related lncRNAs at the cellular and molecular levels in tumors. Based on the public databases, 22 m7G-related lncRNAs were screened out by bioinformatics and statistical analysis of HCC patients’ clinical data. Interestingly, all selected lncRNAs were with high expressions in HCC patients as risk factors. Then, HCC patients were divided into two clusters according to different expressions of lncRNAs. Patients in Cluster 2 had advanced clinical features and worse OS than Cluster 1. CIBERSORT analysis also showed that the effect of immunotherapy might be better in Cluster 1. All these findings indicated that the tumor immune microenvironment has enhanced the oncogenesis of HCC.

In our study, a total of nine out of 22 m7G-related lncRNAs, which included MKLN1-AS, KDM4A-AS1, AC026412.3, SNHG4, AC026356.1, AL031985.3, POLH-AS1, TMCC1-AS1, and LINC01224, were identified as key lncRNAs with HCC patients’ OS. Among them, high MKLN1-AS expression was previously reported to aggravate hepatocellular carcinoma progression and was associated with shorter overall survival and disease-free survival in patients with HCC (Gao et al., 2020). Of these lncRNAs, POLH-AS1 has not been studied but was linked to the development and occurrence of diseases (Zhang et al., 2022b). It is worth noting that the coefficient value of POLH-AS1 levels was the second highest in our constructed model, after MKLN1-AS. The study by Chen al. showed that KDMAS-1 was significantly increased in hepatocellular carcinoma tissues, and the higher the expression of KDMAS-1 was, the worse the prognosis HCC patients had (Bray et al., 2018). AC026412.3 was a member of the prognostic model of m6A-associated lncRNA conducted by Wang, which was significantly correlated with tumor grade, stage, and T stage (Wang et al., 2021). Several diseases are associated with overexpression of SNHG4, and silencing SNHG4 is expected to be a new therapeutic target for human diseases (Chu et al., 2021). Investigators found that overexpressed SNHG4 was closely related to advanced tumor grade, stage, and OS (Zhu et al., 2019; Jiao et al., 2020). According to Wang, SNHG4 was one of the two lncRNAs highly associated with HCC prognosis in a prognostic model (Wang et al., 2021). An autophagy-related and immune-related lncRNA, AL031985.3, had been shown to predict HCC prognosis (Jia et al., 2020; Kong et al., 2020). Similar to TMCC1-AS1, LINC01224 has been implemented in multiple prognostic models for HCC (Zhao et al., 2018; Deng et al., 2020; Xu et al., 2021). After HCC patients were classified into two risk groups (high-risk and low-risk) based on the constructed risk model, the signature we established could predict the OS of HCC patients in the training, testing, and ICGC sets with high accuracy, according to the survival analysis. Univariate and multivariate Cox regression analyses showed that the m7G-LPS was closely related to the OS of HCC. Additionally, we examined the m7G-LPS stratification in clinicopathological features, and the results indicated that m7G-LPS might be useful for the evaluation of clinical prognosis. In summary, based on the rich biological statistics and experimental validation, we have sufficient reasons to believe that the above nine lncRNAs can predict the survival of HCC patients.

Hepatocellular carcinoma is a typical inflammation-associated tumor, and, thus, there may be complex interactions between cancer cells and multiple other cells in the tumor microenvironment. We then performed GSEA to better realize the biological behaviors mediated by m7G-LPS. The GO function enrichment of high-risk score focusing on adaptive immune functions and cell cycle and ECM receptor interaction was enriched in KEGG analysis, while the low-risk group may be significantly enriched in lipid metabolism, indicating that m7G-LPS may affect the prognosis of patients through immune mechanisms. Increasing evidence has confirmed that the cellular and non-cellular components of the tumor microenvironment may impact the prognosis and treatment of HCC. A previous study of pathological sections of HCC patients showed that differential gene expression in tumor cells and their stroma has important implications for tumor survival and molecular subtypes (Hu and Huang, 2022). Recently, immunotherapy provided advanced HCC patients with more options beyond targeted drugs. Immune checkpoint inhibitors clear tumor cells by altering the tumor immune microenvironment so that tumor cells are specifically recognized. In the high-risk group, 33 immune checkpoints were significantly overexpressed compared with the low-risk group. The TIDE algorithm can predict clinical response to immune checkpoint blockade using tumor transcriptomic signatures. Our research discovered that high-risk patients had lower TIDE scores in contrast to those with low-risk scores. All these results suggested that high-risk HCC patients may have a better prospect for immunotherapy.

Hepatocellular carcinoma, due to its heterogeneity, is recognized as one of the most chemotherapy-resistant tumors (Huang et al., 2020; Xu et al., 2020), indicating that different patients may show different responses to conventional treatments, so it becomes crucial to predict the effectiveness of different interventional regimens on HCC patients. Among the five common chemotherapy drugs, we found the high-risk group was more sensitive to axitinib, dasatinib, erlotinib, and sorafenib, while sunitinib may have a better effect in patients in the high-risk group. This suggested that different treatment strategies could be used for patients with different molecular subtypes of hepatocellular carcinoma, providing several insights into the development of precision oncology.

As we all know, immunotherapy lacks efficacy predictors due to its complex mechanism of action. Recently, TMB has been widely explored as a predictive biomarker in some cancers on immunotherapy efficacy (Hellmann et al., 2018; Tang et al., 2021). In the present study, HCC patients with high-risk scores presented a considerably higher TMB than those with low-risk scores. Published research studies reported that patients with high TMB have a worse OS in HCC (Cao et al., 2019). The results were consistent with the present study.

Within our perception, this is the first study to investigate the correlation between m7G-related lncRNAs and HCC pathogenesis in the immune microenvironment and to establish a prognostic model based on public databases. There are, however, several innate limitations in our research. First, the conclusions we had drawn above were based on public databases. Despite abundant biological information being used to elucidate the m7G-related lncRNA, further studies are needed to support these conclusions in a larger HCC patient cohort. More importantly, despite our growing understanding of m7G and lncRNAs, more experiments are required to illustrate how these m7G-related lncRNAs affect HCC.

## Conclusion

The m7G-LPS model had promising prediction performance for OS in HCC. Our results provided comprehensive evidence for further studies indicating the function of m7G-related lncRNAs in HCC, which could shed new perspectives on the tumorigenesis and guidance of tumor immunotherapy in HCC.

## Data Availability

The original contributions presented in the study are included in the article/Supplementary Material; further inquiries can be directed to the corresponding authors.
