# Supplementary material for: Comprehensive analysis of nine m7G-related lncRNAs as prognosis factors in tumor immune microenvironment of hepatocellular carcinoma and experimental validation
Source: Front Genet. 2022 Aug 23;13:929035. doi: 10.3389/fgene.2022.929035 (PMC9445240; doi:10.3389/fgene.2022.929035)
Supplement: Supplementary file 1 [file Table1.DOCX]

| Variable | TCGA(n,%) | ICGC(n,%) |
| --- | --- | --- |
| Age  ≤65  >65 | 232(62.7%)  138(37.3%) | 89(38.7%)  141(61.3%) |
| Gender  Female  Male | 121(32.7%)  249(67.3%) | 61(26.5%)  169(73.5%) |
| Stage  Stage I-II  Stage III-IV  Unknown | 256(69.1%)  90(24.3.%)  24(6.6%) | 142(61.7%)  88(38.3) |
| Grade  G1-2  G3-4  Unknown | 232(62.7%)  133(35.9%)  5(1.4%) | NA |
| T  T1-2  T3-4  Unknown | 274(74.1%)  93(25.1%)  3(0.8%) | NA |
| MVI  Yes  No  Unknown | 88(23.8%)  262(70.8%)  20(5.4%) | NA |

**SUPPLEMENTARY TABLE S1** Clinical Characteristics of the HCC Cases in TCGA and ICGC
