# Supplementary figures and images for: Comprehensive analysis of nine m7G-related lncRNAs as prognosis factors in tumor immune microenvironment of hepatocellular carcinoma and experimental validation
Source: Front Genet. 2022 Aug 23;13:929035. doi: 10.3389/fgene.2022.929035 (PMC9445240; doi:10.3389/fgene.2022.929035)

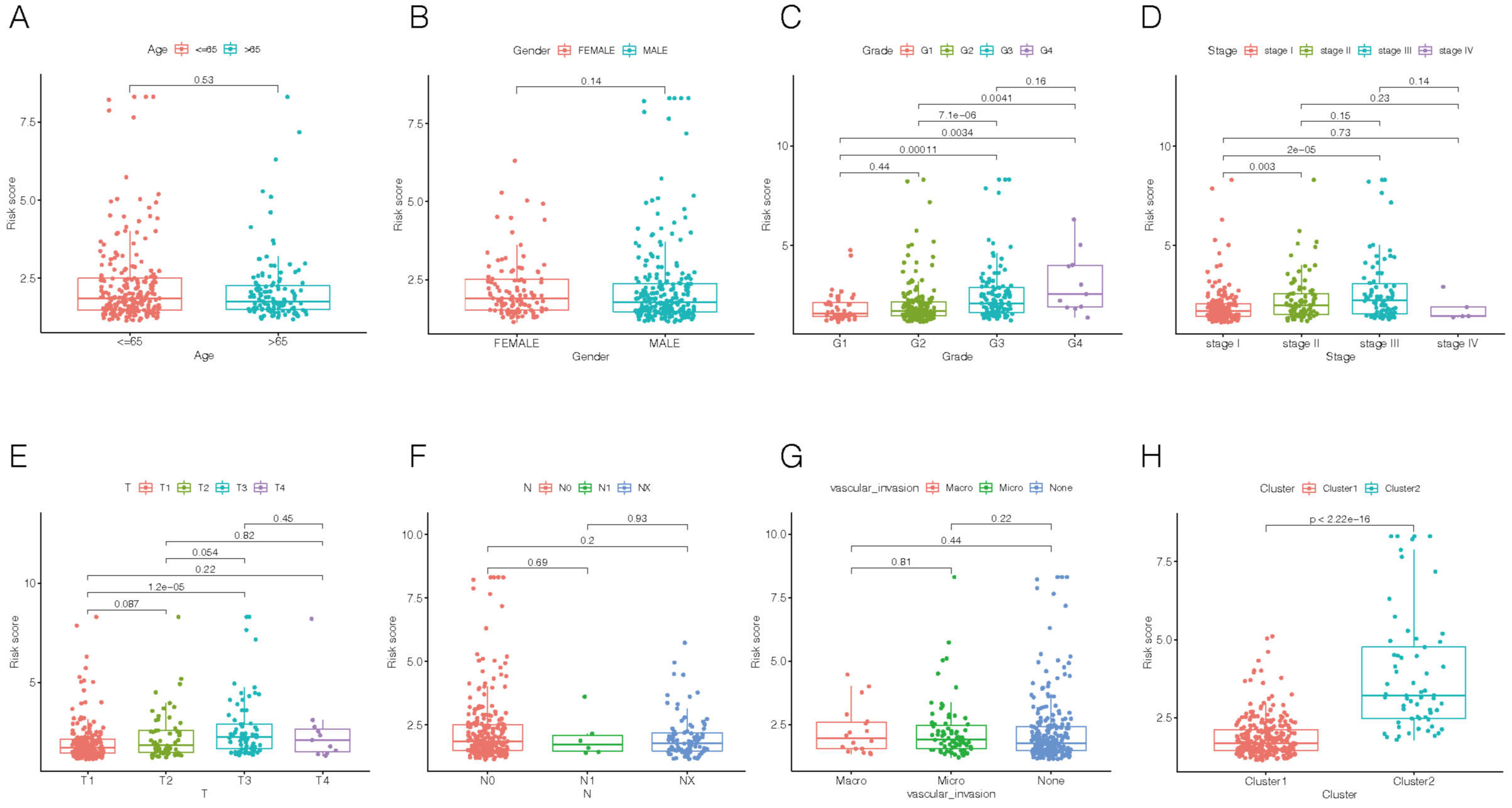

Supplement: Supplementary file 2 [file Image3.TIF]

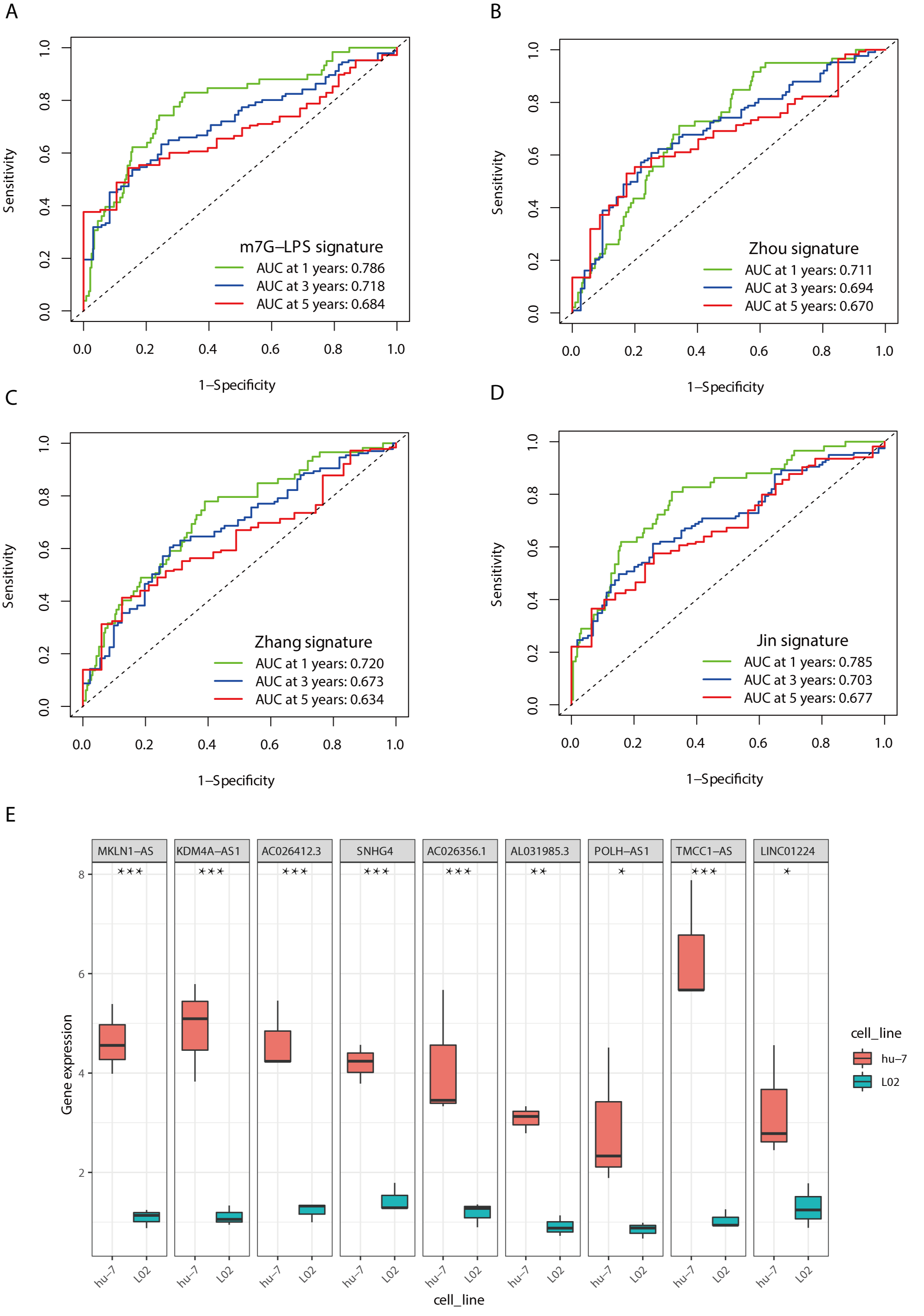

Supplement: Supplementary file 3 [file Image2.TIF]

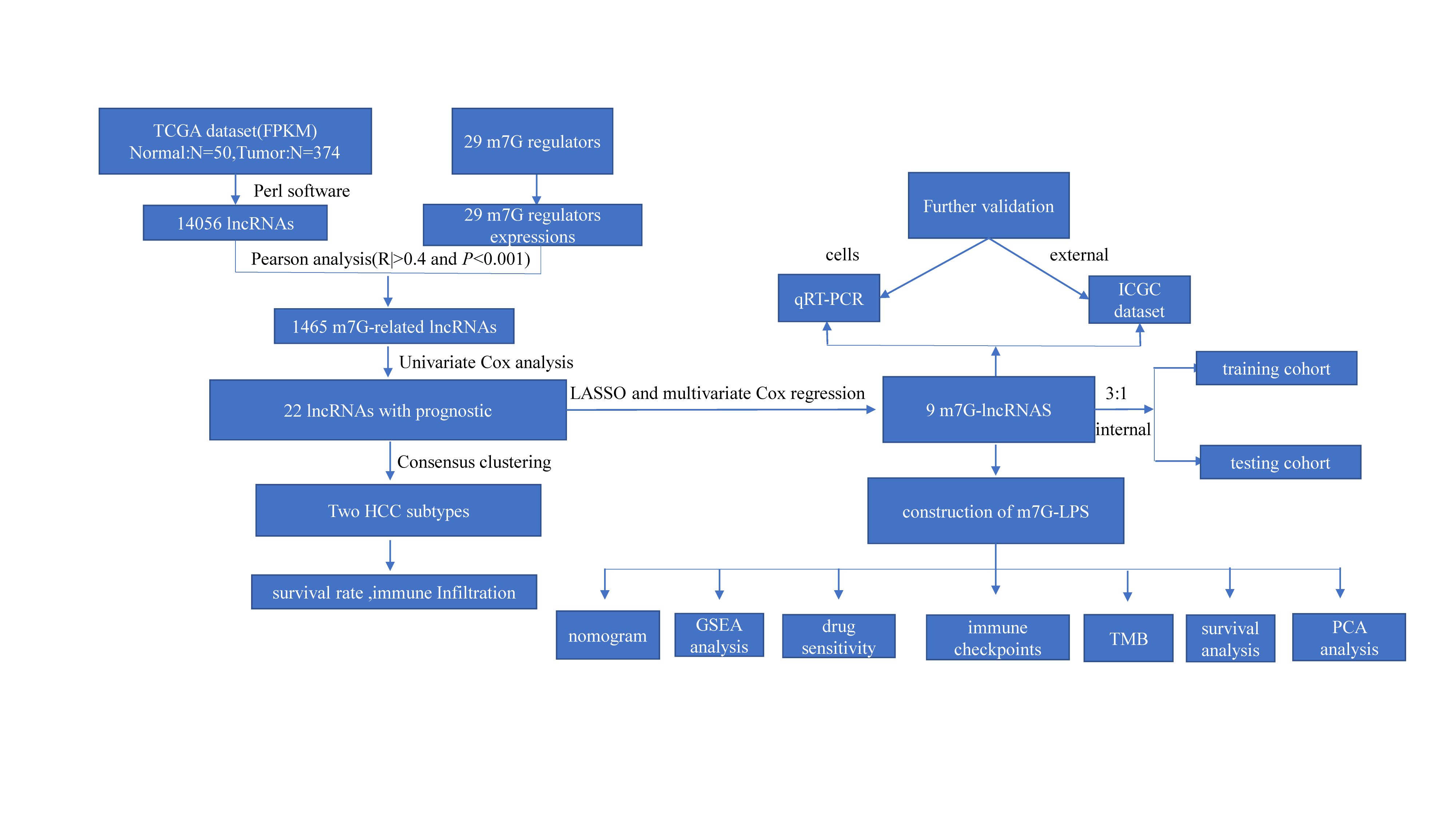

Supplement: Supplementary file 4 [file Image1.TIF]
