# Supplementary material for: Comprehensive analysis of nine m7G-related lncRNAs as prognosis factors in tumor immune microenvironment of hepatocellular carcinoma and experimental validation
Source: Front Genet. 2022 Aug 23;13:929035. doi: 10.3389/fgene.2022.929035 (PMC9445240; doi:10.3389/fgene.2022.929035)
Supplement: Supplementary file 5 [file Table2.DOCX]

**SUPPLEMENTARY TABLE S2 |** Details of twenty-nine m7G regulators.

| **Gene symbol** | **Full name** |
| --- | --- |
| METTL1 | methyltransferase 1 |
| WDR4 | WD repeat domain4 |
| NSUN2 | NOP2/Sun RNA methyltransferase 2 |
| DCP2 | Decapping MRNA 2 |
| DCPS | Decapping Enzyme, Scavenger |
| NUDT10 | Nudix Hydrolase 10 |
| NUDT11 | Nudix Hydrolase 11 |
| NUDT16 | Nudix Hydrolase 16 |
| NUDT3 | Nudix Hydrolase 3 |
| NUDT4 | Nudix Hydrolase 4 |
| NUDT4B | Nudix Hydrolase 4B |
| AGO2 | Argonaute RISC Catalytic Component 2 |
| CYFIP1 | Cytoplasmic FMR1 Interacting Protein 1 |
| EIF4E | Eukaryotic Translation Initiation Factor 4E |
| EIF4E1B | Eukaryotic Translation Initiation Factor 4E Family Member 1B |
| EIF4E2 | Eukaryotic Translation Initiation Factor 4E Family Member 2 |
| EIF4E3 | Eukaryotic Translation Initiation Factor 4E Family Member 3 |
| GEMIN5 | Gem Nuclear Organelle Associated Protein 5 |
| LARP1 | La Ribonucleoprotein 1, Translational Regulator |
| NCBP1 | Nuclear Cap Binding Protein Subunit 1 |
| NCBP2 | Nuclear Cap Binding Protein Subunit 2 |
| NCBP3 | Nuclear Cap Binding Subunit 3 |
| EIF3D | Eukaryotic Translation Initiation Factor 3 Subunit D |
| EIF4A1 | Eukaryotic Translation Initiation Factor 4A1 |
| EIF4G3 | Eukaryotic Translation Initiation Factor 4 Gamma 3 |
| IFIT5 | Interferon Induced Protein With Tetratricopeptide Repeats 5 |
| LSM1 | LSM1 Homolog, MRNA Degradation Associated |
| NCBP2L | Nuclear Cap Binding Protein Subunit 2 Like |
| SNUPN | Snurportin 1 |
