# Supplementary material for: Comprehensive analysis of nine m7G-related lncRNAs as prognosis factors in tumor immune microenvironment of hepatocellular carcinoma and experimental validation
Source: Front Genet. 2022 Aug 23;13:929035. doi: 10.3389/fgene.2022.929035 (PMC9445240; doi:10.3389/fgene.2022.929035)
Supplement: Supplementary file 6 [file Table3.DOCX]

**Supplement Table S3** The primer sequences of nine m7G-related lncRNAs.

| Gene name | Sequence of primer |
| --- | --- |
| MKLN1-AS | F:AAAGAGTATGTCGCTTATTGTCTAAGA |
|  | R: ATCCTGCTGACTTACTCCAGATGT |
| KDM4A-AS1 | F:TTGCCTGGATGGCTGAGAATC |
|  | R:TTCCTTTCACCCTCCTTCCTT |
| AC026412.3 | F: TGTGAGGTGAGGGAGCGAT |
|  | R: TGAGCCAAAGGGATCTACGC |
| SNHG4 | F: GCAGGTGACAGTCTGCATGT |
|  | R: TTTTAAGTCCCCTACCCCCATC |
| AC026356.1 | F: GGTGACTCAGAACGTCTCCTC |
|  | R: GGGGGCCTGCAATCTACAAG |
| AL031985.3 | F: TGTGGTCCCTGTCACACCTA |
|  | R: AGAAGCCAAGGATTCCCCTA |
| PLOH-AS1 | F:TTTGTCTCTGCGGAGGTTCC |
|  | R:GGCAGGTGGATCATTTGAGGTCAG |
| TMCC1-AS1 | F: AGCGAGGGATCGAGTTGAGA |
|  | R: AGCGAGGGATCGAGTTGAGA |
| LINC01224 | F: AGAGCTTGGGATCGCTTTCTG |
|  | R: TTACTCAGGTGCCTTTCCCAC |
